# 2D-WinSpatt-Net: A Dual Spatial Self-Attention Vision Transformer Boosts Classification of Tetanus Severity for Patients Wearing ECG Sensors in Low- and Middle-Income Countries

**DOI:** 10.3390/s23187705

**Published:** 2023-09-06

**Authors:** Ping Lu, Andrew P. Creagh, Huiqi Y. Lu, Ho Bich Hai, VITAL Consortium, Louise Thwaites, David A. Clifton

**Affiliations:** 1 Department of Engineering Science, University of Oxford, Oxford OX1 3PJ, UK; 2 Oxford University Clinical Research Unit, Ho Chi Minh City 700000, Vietnam; 3 Oxford Suzhou Centre for Advanced Research, Suzhou 215123, China

**Keywords:** tetanus, continuous wavelet transform, electrocardiogram, classification, attention, transformer, time series imaging

## Abstract

Tetanus is a life-threatening bacterial infection that is often prevalent in low- and middle-income countries (LMIC), Vietnam included. Tetanus affects the nervous system, leading to muscle stiffness and spasms. Moreover, severe tetanus is associated with autonomic nervous system (ANS) dysfunction. To ensure early detection and effective management of ANS dysfunction, patients require continuous monitoring of vital signs using bedside monitors. Wearable electrocardiogram (ECG) sensors offer a more cost-effective and user-friendly alternative to bedside monitors. Machine learning-based ECG analysis can be a valuable resource for classifying tetanus severity; however, using existing ECG signal analysis is excessively time-consuming. Due to the fixed-sized kernel filters used in traditional convolutional neural networks (CNNs), they are limited in their ability to capture global context information. In this work, we propose a 2D-WinSpatt-Net, which is a novel Vision Transformer that contains both local spatial window self-attention and global spatial self-attention mechanisms. The 2D-WinSpatt-Net boosts the classification of tetanus severity in intensive-care settings for LMIC using wearable ECG sensors. The time series imaging—continuous wavelet transforms—is transformed from a one-dimensional ECG signal and input to the proposed 2D-WinSpatt-Net. In the classification of tetanus severity levels, 2D-WinSpatt-Net surpasses state-of-the-art methods in terms of performance and accuracy. It achieves remarkable results with an F1 score of 0.88 ± 0.00, precision of 0.92 ± 0.02, recall of 0.85 ± 0.01, specificity of 0.96 ± 0.01, accuracy of 0.93 ± 0.02 and AUC of 0.90 ± 0.00.

## 1. Introduction

The life-threatening infectious disease tetanus is prevalent in low- and middle-income countries (LMIC); although this disease is unusual in high-income countries, it continues to be seen in these settings [1,2,3]. Tetanus is caused by a bacterium called *Clostridium tetani* [4]. Despite the availability of tetanus vaccinations and antitoxin for acute treatment, an estimated 213,000 to 293,000 tetanus patients die worldwide each year [5].

Tetanus toxin hinders the transmission of signals at synapses within the central nervous system, leading to painful muscle spasms and stiffness. Cardiovascular system instability occurs in severe cases due to the toxin effect in the autonomic nervous system (ANS). Over a period of 2 to 5 days, approximately 50% of patients will advance to severe disease, and, in the absence of treatment, muscle spasms impede the ability to breathe. These patients need strong muscle relaxants to counteract spasms and mechanical ventilation to support breathing. Approximately a quarter of all tetanus patients encounter ANS dysfunction, which causes blood pressure and heart rate instability. This ANS dysfunction is the primary cause of mortality among tetanus patients in facilities equipped with mechanical ventilation. However, managing this condition remains challenging. The prompt detection of severe tetanus at its early stages is extremely helpful, because it allows prompt intervention and facilitates more efficient allocation of resources [6]. The Ablett score is extensively employed for the tetanus severity classification system and spans from 1 to 4 [2], allocating a severity grade based on impact on the respiratory and cardiovascular systems. Patients experiencing mild or moderate tetanus (grades 1 and 2) can be managed through non-invasive clinical approaches. Patients with severe tetanus (grades 3 and 4) require full intensive care unit (ICU) care, including mechanical ventilation. For patients with severe tetanus (grade 4), extra organ support may be required to address the effects of ANS involvement. The conventional Ablett grading system relies on a combination of clinical characteristics (e.g., tachycardia, fever and hypertension). In clinical settings with high patient volumes or limited staffing experience, achieving precise classification can be challenging.

Advanced continuous monitoring systems in the intensive and high staff-to-patient ratios in high-income countries are associated with enhanced tetanus outcomes [7,8]. However, the cost of delivering ICU treatment is high across all nations, including LMIC. Furthermore, in LMIC, inadequate equipment and limited time are also frequently mentioned as obstacles in delivering superior care to patients affected by tetanus.

In most limited-resource settings, close monitoring and timely emergency treatment are frequently only available in high-dependency wards or ICUs, as these facilities possess the necessary staff and equipment to provide such services. This large burden of additional cases results in suboptimal use of already limited resources and potentially leads to poorer outcomes for individuals in need of intensive care [7,9,10]. Furthermore, numerous patients in LMIC (e.g., Vietnam) bear the out-of-pocket medical expenses, and the additional costs associated with ICU care are considerably higher in comparison to standard ward care. Previous research has provided information about the direct medical costs for ICU patients with tetanus, dengue and sepsis in Vietnam [7,9,10].

Affordable wearable sensors have been suggested as a viable alternative approach for tetanus in settings with limited resources. The wearable sensors operate wirelessly and are small and lightweight. These sensors can provide real-time, continuous monitoring of vital signs, with the aim of facilitating the early detection of patient deterioration [7,11]. Our previous work has shown that electrocardiogram (ECG) monitoring alone can be used to classify the severity of tetanus [12,13]. Using affordable wearable sensors is still challenging due to inherent inaccuracies in the collected continuous physiological data. This is mainly attributed to missing data and the substantial amount of noise generated by various factors, diminishing its reliability [7].

This study employs ECG data obtained from wearable sensors utilised in an ICU in Vietnam and suggests a rapid triage tool, developed through deep learning techniques, to categorize tetanus severity based on the Ablett score. We design a dual self-attention Vision Transformer named 2D-WinSpatt-Net. The proposed 2D-WinSpatt-Net outperforms the previous methods of 1D and 2D convolution neural network (CNN), and 2D CNN with different attention mechanisms (e.g., 2D-CNN + Channel-wise Attention Network [12]), ViT and a hybrid CNN-Transformer Network [13]. We investigate the time series imaging—continuous wavelet transform (CWT)—as the input for the 2D-WinSpatt-Net. Moreover, we show the difference in generating the CWT and log-spectrogram image based on the tetanus ECG data and discuss why CWT works better in the proposed 2D-WinSpatt-Net. This study provides the following contributions:

- We propose a novel dual self-attention Vision Transformer model that contains both the local spatial window attention and global spatial attention mechanisms on the image patch token level rather than the image pixel level. The local spatial window attention works on the image patches, which obtain the fine-grained features and reduces the complexity to linear. Then the global spatial attention works on the output of the local spatial window attention, telling the proposed model where to look and focus.
- The resized and stitched time series imaging—continuous wavelet transform (CWT)—is explored for the first time to represent the tetanus ECG information. We can obtain better accuracy of tetanus severity level classification using shorter tetanus ECG (20-s), compared to 60-s ECG in previous work on tetanus infectious diseases.
- The proposed 2D-WinSpatt-Net surpasses the performance of the state-of-the-art methods in tetanus classification. It can assist clinical decision making in resource-limited settings.

**Figure 1:**
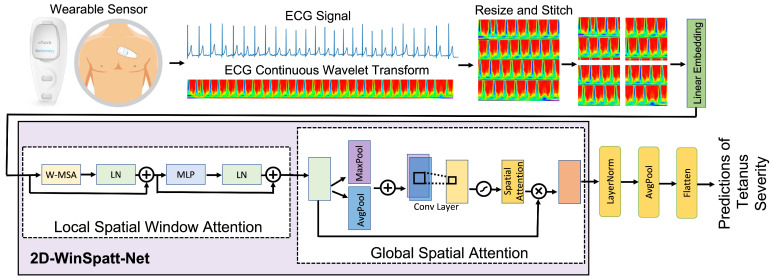
Framework overview for tetanus severity classification. The ePatch wearable sensor is used to acquire raw ECG data. The proposed method, named 2D-WinSpatt-Net, takes the resized and stitched continuous wavelet transform (CWT) of the raw ECG data, with a window length of 20-s, as its input. The output of this method is a label classification, with label 0 representing mild tetanus and label 1 representing severe tetanus.

The structure of the paper is as follows: Section 2 presents an overview of related work in the tetanus diagnosis in LMIC, time series imaging and machine learning techniques. Section 3 outlines the proposed 2D-WinSpatt-Net network (see Figure 1). Section 4 provides comprehensive information about the collected tetanus dataset, implementation specifics, a comparison of baseline methods and evaluations. Section 5 and Section 6 present the results and discuss the experimental findings. Finally, Section 7 delivers the final conclusions drawn from our research.

## 2. Related Work

The severity level of tetanus infection is linked to the functioning of the ANS [12]. Heart rate variability (HRV) measures the fluctuations in the time intervals between consecutive heartbeats (RR intervals). The HRV variations are regulated by the ANS and serve as an indicator of ANS activity [12]. Alterations in conventional HRV parameters obtained from electrocardiography (ECG) have been demonstrated to be associated with the severity of tetanus infection. To classify tetanus severity, HRV-based methods require an additional pre-processing stage for extracting RR intervals and QRS complex [14,15,16,17]. The conventional techniques for detecting HRV require high-cost equipment and expertise, making them usually inaccessible in ICUs or limited-resource settings. Van et. al. [7] suggested extracting RR intervals from tetanus ECG data using wearable devices. However, it remains a persistent challenge to reliably extract accurate RR intervals [18].

Healthcare has undergone a profound transformation with artificial intelligence, encompassing machine learning (ML) and deep learning (DL) techniques [19]. Conventional ML methods require the manual extraction of features. For instance, RR intervals are extracted from the dataset [20]. The support vector machine (SVM) is applied to automatically identify the degree of ANS dysfunction in tetanus [21]. DL methods have demonstrated superior performance compared to traditional machine-learning techniques such as SVM [18]. The experimental results of previous research were limited, because of the small datasets, which contain synchronised physiological data obtained from a group of 10 patients diagnosed with tetanus [18,21] and PPG data collected from 19 tetanus patients [19]. In our most recent study [12,13], ECG data were collected from 110 tetanus patients, using the low-cost wearable monitor.

Time series imaging is a technique that converts temporal data into visual representations, commonly employed in 2D convolutional neural networks (CNNs) for classification purposes [12,13,18,22,23,24,25]. Time series imaging can be gramian angular field, recurrence plot, spectrogram or continuous wavelet transform [26]. One-dimensional (1D) convolutional neural networks (CNNs) have been utilised in various biomedical signal processing tasks, including the classification of biomedical data and the early detection of medical conditions [27,28]. However, an image-based ECG signal classification structure using time series imaging (2D spectrograms) surpasses the performance of traditional 1D CNN models [29]. Utilising spectrograms, transfer learning and a combination of ECG and PPG data, researchers have successfully employed these techniques to classify the severity of two infectious diseases: HFMD and Tetanus [18]. Lu et al. [12,13] suggested the logarithmic spectrogram represented the ECG signal. Results showed that the image-based ECG signal classification networks, the 2D-CNN-Transformer/8 [13] and the 2D-CNN + Channel-wise Attention Network [12] achieve better performances than the 1D CNN.

Transformer [30] is remarkable for capturing global or long-range dependencies through parallel self-attention mechanisms. This has proven to be highly effective in a wide range of natural language processing (NLP) tasks. The remarkable achievements observed in the field of NLP using the Transformer model have inspired researchers to explore its application in the domain of computer vision [31]. Vision Transformer (ViT) [32] is an extension of Transformer, which already surpasses all previous benchmarks and achieves the state-of-the-art technique in image classification. An input image is split into a set of 16×16 non-overlapping image patches, named visual tokens. Next, these patches are combined with positional encoding and fed into transformer blocks to capture global relationships for the classification. Multiple variations of Vision Transformers (ViTs) have been proposed with the aim of enhancing performance in vision tasks. For instance, Swin Transformer is a hierarchical ViT choosing shifted windows [33], which achieved better performances than the ViT and CNN-based architectures. Data-efficient image Transformer (DeiT) [34] employs knowledge distillation for image classification [35]. TNT [36] processes the relationship between sub-patches via an inner transformer block and captures the interconnections among patch-level embeddings via an outer transformer block.

Transformers have emerged as a significant breakthrough in the field of computer vision and image analysis [13,37,38,39,40,41]. Our previous work [13] is the initial implementation of a transformer-based method for categorizing tetanus severity levels, which can help to triage patients quickly in LMIC wearing ECG sensors. The hybrid CNN-Transformer Network [13] is inspired by transformers on audio spectrograms [42,43,44]. Tetanus ECG is represented by a log-spectrogram. The ViT Encoder is employed in this hybrid CNN-Transformer Network. Transformers have great potential for stratifying tetanus severity levels, which has not been fully investigated. Hence, we need to explore further methodology based on Transformers.

## 3. Method

### 3.1. Data Preprocessing

During the pre-processing step, the crucial objective is to denoise an ECG signal. There are two primary types of noise—low-frequency noise [45] and high-frequency noise [45]— which disturb the ECG signal analysis. The presence of low-frequency noise arises from patient muscle movement, while high-frequency noise stems from the electrical source that powers the ECG monitor.

In this study, we obtain one-lead ECG signals from an affordable wearable monitor. To enhance the data quality, we employ a Butterworth filter to eliminate background noise and refine the signals. The high-pass filter is set at a cutoff frequency of 0.05 Hz, while the low-pass filter is set at a cutoff frequency of 100 Hz. We utilise the SciPy package [46] to implement the data preprocessing step.

### 3.2. Continuous Wavelet Transform

In this work, we visualised the ECG waveform in its time–frequency representation using (discrete) continuous wavelet transform (CWT). CWT is a technique employed to assess the similarity between a signal and an analysing function, enabling a refined depiction of the signal’s time–frequency characteristics [47,48], as compared to computing a spectrogram with consecutive Fourier transforms over windowed time. The CWT of a discrete time signal, $x_{n}$, with a constant sampling period, $\delta_{t}$, can be expressed as the outcome of convolving $x_{n}$ with a mother wavelet that has been scaled and translated.

(1a) $$W_{n}\left(s\right)=\sum_{n^{\prime }=0}^{N-1}x_{n^{{}^{\prime }}}\psi {}^{*}\left[\frac{\left(n^{{}^{\prime }}-n\right)\delta_{t}}{s}\right]$$

where (${}^{*}$) denotes the complex conjugate, *s* is the wavelet scaling factor and *n* is the localised time index. The subscript ${}_{0}$ on ψ has been dropped to indicate that this $\psi_{0}$ has been multiplied by ${\left(\frac{\delta_{t}}{s}\right)}^{1/2}$, in order to normalise ψ to have unit energy. This ensures that the wavelet transforms, $W_{n}\left(s\right)$, at each scale, *s*, are directly comparable to each other and to the transforms of other time series; see [49]. In this work, we used a Morlet mother wavelet, which has previously been shown to effectively capture the morphology of various biomedical signals, including ECG [26,50,51,52]. A Morlet wavelet consists of a plane wave modulated by a Gaussian:

(1b) $$\psi_{0}\left(\eta \right)=\frac{1}{\sqrt[{4}]{\pi }}e^{i\cdot w_{0}\cdot \eta }\cdot e^{\frac{-\eta^{2}}{2}}$$

where η is a non-dimensional time parameter and $w_{0}$ is the non-dimensional frequency, here taken to be 6, as per [49], to satisfy the admissibility condition. The total signal energy at a specific scale can be measured by the scale-dependent energy density spectrum, $E_{s}$:

(1c) $$E_{s}=\sum_{n=0}^{N-1}{\left|W_{n}\left(s\right)\right|}^{2}$$

where s∈[1,S] and $|W_{n}{\left(s\right)|}^{2}$ represent the scalogram, a 2-D wavelet energy density that captures and quantifies the complete energy distribution of the signal.

The frequency, *f*, in Hz, can be approximated from the wavelet scaling factor, *s*, such that [53]:

(2a) $$f=\frac{f_{c}}{s}$$

where the center frequency in Hz can be defined by [49]:

(2b) $$f_{c}=\frac{w_{0}+\sqrt{2+w_{0}^{2}}}{4\pi }$$

**Figure 2:**
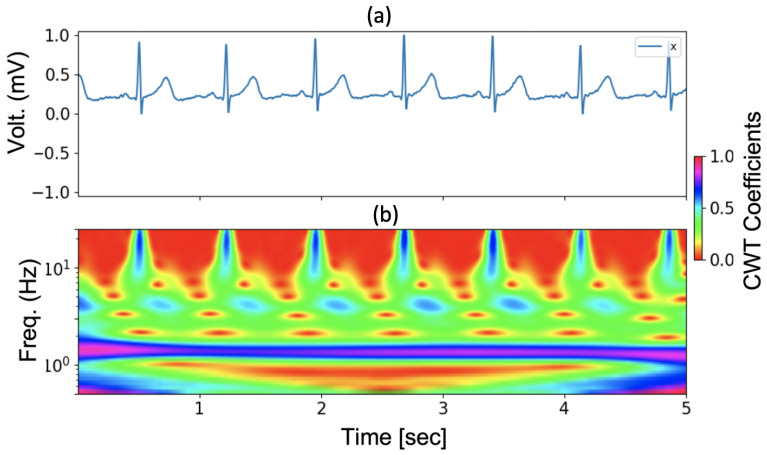
An example of tetanus ECG and continuous wavelet transform (CWT): (**a**) Tetanus ECG in 5-s; (**b**) The CWT related to (**a**).

Figure 2 shows an example of a tetanus ECG (in 5 s) and the subsequent time–frequency resolution using CWT.

### 3.3. 2D-Winspatt-Net

#### 3.3.1. Preliminaries

We first introduce the basic components in 2D-WinSpatt-Net, including multi-layer perceptron (MLP), layer normalization (LN) and window-based multi-head self-attention (W-MSA).

**MLP.** The MLP means multi-layer perceptron or multiple fully connected layers, which can be described as MLP(X)=FC(σ(FC(X))), where σ(.) represents an activation function GELU [54]; the FC means a fully-connected layer.

**LN.** Layer normalization [55] enhances the stability of hidden state dynamics within the training network, resulting in expedited training time and improved convergence. The equation is given by

(3) $$LN\left(x\right)=\gamma \circ \frac{x-\mu }{\varrho }+\beta ,$$

where μ and ϱ are the average value and standard deviation of the elements in *x*, γ and β are learnable parameters and ∘ represents the element-wise dot.

**W-MSA**. The attention is calculated within each window, which is different from the standard MSA. In our previous work [13], we chose the standard MSA to compute global self-attention. The global self-attention considers the relationship between each patch in an image. Each patch is compared to all other patches in an image. However, the computational cost increases remarkably when the size of the image grows. If the window size is fixed, the complexity of window-based MSA is linear, with the number of patches based on the size of the image.

In a local window with M×M patches, a group of relative position bias $B=\{B_{i}\in R^{M_{i}^{2}\times M_{i}^{2}},i=1,2,\ldots ,n_{win}\}$ is added to compute the similarity of each head of W-MSA. In the i-th local window, the W-MSA can be described as

(4) $$Att\left(Q_{i},K_{i},V_{i}\right)=softmax\left(\frac{Q_{i}K_{i}^{T}}{\sqrt{d}}+B_{i}\right)V_{i},$$

where $Q_{i}\in R^{M_{i}^{2}\times d}$, $K_{i}\in R^{M_{i}^{2}\times d}$ and $V_{i}\in R^{M_{i}^{2}\times d}$ are queries, keys and values, and $M_{i}^{2}$ is the number of patches in the i-th local window. The scale factor $\frac{1}{\sqrt{d}}$ leads to stable gradients.

#### 3.3.2. Local Spatial Window Attention

Given an input CWT image $x\in R^{W\times H\times C}$, where the *C* represents the channel quantity and *W* and *H* indicate the width and height of the feature map, we first split the x into flattened non-overlapping patches. Each patch is treated as a “token” and its feature is set as a concatenation of the raw pixel RGB values. We consider each patch as a “token” and represent its feature by combining the raw pixel RGB values into a concatenated form. The raw-valued feature undergoes a linear embedding process that maps it to a vector of an arbitrary dimension, *D*. Secondly, the windows are organised to partition these image patches evenly. The local spatial window-based attention works on these patch tokens, and it is calculated within each local window. In our work, we use a patch size of 4 × 4. The window-based attention module maintains the number of tokens $(\frac{H}{4}$×$\frac{W}{4})$.

After the embeddings, we employ L transformer layers. The output of the l-th layer is as follows:

(5) $$m_{l}^{\prime }=LN\left(W-MSA\left(m_{l-1}\right)\right)+m_{l-1},\;\;\;\;l=1,\ldots ,L,$$

(6) $$m_{l}=LN\left(MLP\left(m_{l}^{\prime }\right)\right)+m_{l}^{\prime },\;\;\;\;l=1,\ldots ,L.$$

The $m_{l}$ denotes the output features of the W-MSA module and the MLP module for the l-th layer after LN operation. In our implementation, one transformer layer can achieve an optimal result.

We assume each local window with M×M patches; the computational complexity of a global MSA module and a window-based W-MSA module on the image of h×w patches are as follows:

(7) $$\Omega \left(MSA\right)=4hwD^{2}+2{\left(hw\right)}^{2}D,$$

(8) $$\Omega \left(W-MSA\right)=4hwD^{2}+2M^{2}hwD,$$

where the global self-attention computation is quadratic to patch number, hw, and the window-based W-MSA is linear when *M* is fixed (the default value is 7). Global self-attention computation is too expensive for a large hw, while the window-based self-attention offers scalability. The W-MSA computation is reduced compared to standard global MSA. However, the window-based self-attention module does not have connections across windows, which forfeits the capacity to model the global information. In order to deal with this challenge, we propose a global spatial attention after the local spatial window attention module.

#### 3.3.3. Global Spatial Attention

A simple and effective attention module is designed to boost the performance of convolutional neural networks (CNNs), such as squeeze and excitation (SE) block [56] and the convolutional block attention module (CBAM) [57]. Inspired by these attention modules in CNNs, we build a spatial attention map based on the inter-spatial relationship of outcome features of local spatial window attention. To calculate the spatial attention, we initially perform global average-pooling, $F_{\mathrm{AvgPool}}\left(m_{l}\right)$, and global max-pooling, $F_{\mathrm{MaxPool}}\left(m_{l}\right)$, operations along the channel axis and then concatenate them to produce an efficient feature vector. Next, we generate a spatial attention map, $M_{\mathrm{spatial}}\left(m_{l}\right)$, using a convolution layer on the concatenate feature vector.

(9) $$m_{l}=M_{spatial}\left(m_{l}\right).$$

(10) $$M_{spatial}\left(m_{l}\right)=\sigma \left(f^{7\times 7}\left(\left[F_{AvgPool}\left(m_{l}\right);F_{MaxPool}\left(m_{l}\right)\right]\right)\right),$$

where σ represents the sigmoid function and $f^{7\times 7}$ means a convolution operation with the filter size of 7×7.

## 4. Experiments

### 4.1. ECG Data from Tetanus Patients

The collection of tetanus data has obtained approval from both the Oxford Tropical Research Ethics Committee and the Ethics Committee of the Hospital for Tropical Diseases. This dataset is obtained from the Hospital for Tropical Diseases, located in Ho Chi Minh City, Vietnam. This tetanus dataset was published in 2021 [7].

For our study, we utilised ECG data obtained from patients diagnosed with tetanus. The ePatch, a low-cost wearable monitor, was chosen as the monitoring device (ePatch V.1.0, BioTelemetry, Malvern, PA, USA) (see Figure 1). The 7g-weight ePatch (ePatch. https://www.philips.co.uk/healthcare/resources/landing/epatch, accessed on 1 September 2023) sensor was securely pressed onto the patient’s chest skin, ensuring firm adhesion. The ePatch device captures ECG readings in two channels at a sampling rate of 256 Hz.

The two channels (channel 1 and 2) of the ePatch device are not directly correlated with the conventional bedside monitor’s ECG leads 1 and 2. [13]. The recorded continuous ECG data were stored within the ePatch and later exported upon completion of the recording period. The study focused on adult tetanus patients (age ⩾ 16 years), who were admitted to the ICU at the Hospital for Tropical Diseases in Ho Chi Minh City. Collection of vital-sign monitoring data included the recording of two approximately 24-h ECG datasets: one on the day of enrolment (1st day in the ICU) and another on the 5th day of hospitalization. For our experiment, we only utilised ECG signals captured from channel 1 of the ePatch device. To ensure signal stability, we trimmed the initial and final five minutes of each ECG recording [7].

The dataset comprises a total of 178 ECG waveform example files collected from 110 patients during their enrolment and on the 5th day of hospitalization (referred to as days 1 and 5). To ensure data separation, the dataset is divided into training, validation and test sets in a ratio of 141/19/18, respectively. Importantly, the same patient data are not present in multiple sets simultaneously. The time-series ECG waveform is divided into a sequence of non-overlapping ECG samples, with each window length set to 20 s. This duration is shorter than the 60-s window length used in our previous work [12,13].

### 4.2. Implementation Details

**Pre-processing.** From each ECG example file, we selected 30 ECG time series, each lasting 20 s. Consequently, the training set contains a total of 4230 (141 ∗ 30) ECG continuous wavelet transform (CWT) samples, comprising 2370 samples of mild disease and 1860 samples of severe disease. The validation set consists of 540 ECG CWT samples (18 ∗ 30), with 270 samples representing mild disease and 270 samples representing severe disease. Similarly, the test set includes 570 ECG CWT samples (19 ∗ 30), with 360 samples denoting mild disease and 210 samples representing severe disease. The labelling of mild and severe tetanus cases was performed by clinicians at the Hospital for Tropical Diseases. For a detailed overview, please refer to Table 1.

**Figure 3:**
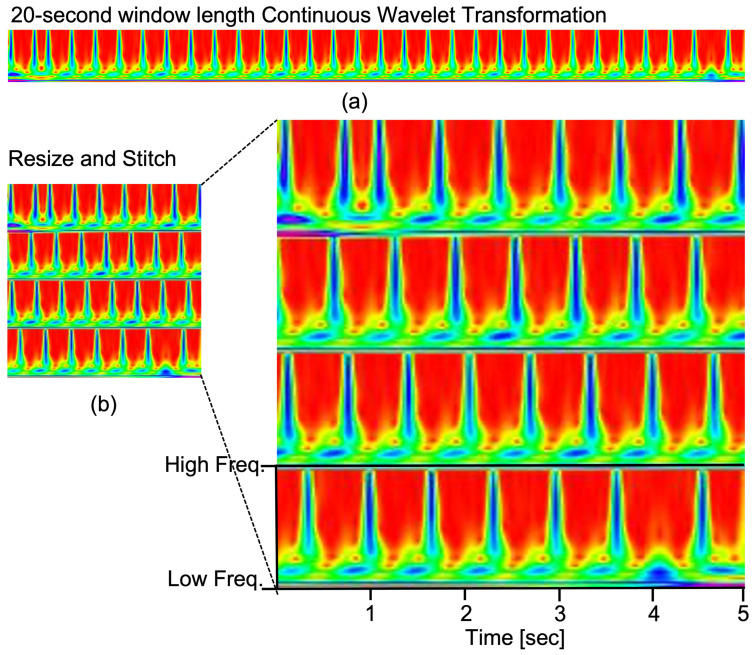
The various approaches for time series imaging include the following: (**a**) Utilising a CWT with a window length of 20 s on images sized 224 pixels ×224 pixels. (**b**) Employing CWT after resizing and stitching the images obtained from (**a**), resulting in images of dimensions 224 pixels ×224 pixels.

Based on our previous experiments [13], we employed a resizing and stitching process to transform the continuous wavelet transform (CWT) into a square image format. The resulting square CWT was saved as a JPG image file, utilizing the default ’hsv’ colour map. This square CWT image represents a resolution of 224×224 pixels, capturing the CWT over every 20 s of ECG data. These processed CWT images are then utilised as input for the proposed 2D-WinSpatt-Net architecture (refer to Figure 3).

**Experimental Setup.** Based on our experiments, the local spatial window attention with the following selected hyperparameters of the proposed 2D-WinSpatt-Net achieves optimal results:

- Image size: 224;
- Input channels: 3;
- Patch size: 4;
- Number of classes: 2;
- Embedding dimension: 96;
- Transformer blocks: 1;
- Number of heads: 2;
- Window size: 7;
- Query, keys and values bias: True;
- MLP ratio: 4.

The model is trained for 100 epochs, employing the Adam optimizer with a learning rate set at 0.001 and a batch size of 32. The torch.nn.BCEWithLogitsLoss is selected as the loss function. The implementation of the suggested 2D-WinSpatt-Net was carried out in Python 3.7 using PyTorch. The experiments were conducted on computational hardware consisting of the NVIDIA RTX A6000 48GB GPU.

### 4.3. Baselines

In our work, we compare the proposed method—2D-WinSpatt-Net—with six different baseline methods. These baseline methods encompass five 2D deep learning approaches, namely 2D-CNN, 2D-CNN + Dual Attention, 2D-CNN + Channel-wise Attention [12], 2D-CNN-Transformer/8 [13] and Swin Transformer, alongside a 1D-CNN method. Furthermore, we evaluate the performance of the proposed 2D-WinSpatt-Net by employing two different types of time series imaging, namely log-spectrogram and continuous wavelet transform (CWT), as input representations.

### 4.4. Evaluation Metrics

In this study, we employed several performance metrics to assess the effectiveness of the binary classification task. These metrics contain F1-score, precision, recall, specificity, accuracy [18] and the area under the curve (AUC) [58]. To ensure robustness, each model was executed five times, and the average and standard deviation of the performance metrics were computed and reported using an independent test dataset. A higher AUC indicates superior model performance in accurately distinguishing between severe and mild cases of tetanus.

The F1-score is a metric that quantifies the balanced combination of precision and recall. A higher F1-score indicates better model performance in precision and recall in classification tasks. The F1-score is defined by the following formula:

(11) $$F1=2*\frac{Precision*Recall}{Precision+Recall}.$$

The precision rate calculates the percentage of true positives among the data that the model predicted as positive. Recall rate represents the model’s ability to correctly identify all positive cases in the data, and it is also called the sensitivity rate. Precision rate is often reported with the recall rate, both useful in evaluating how precisely a method predicts the true positive labels. True positive (*TP*) refers to the accurate prediction of severe tetanus cases, while true negative (*TN*) signifies the correct identification of mild tetanus cases. False positive (*FP*) refers to the instances where mild tetanus is inaccurately classified as severe tetanus, while false negative (*FN*) refers to cases where severe tetanus is inaccurately classified as mild tetanus. Precision and recall are defined as follows:

(12) $$Precision=\frac{TP}{TP+FP}.$$

(13) $$Recall=\frac{TP}{TP+FN}.$$

Specificity measures the percentage of true negatives that a model correctly classifies out of all the negative instances in the data. It measures a model’s ability in correctly identifying all negative instances in the data. The specificity is defined as:

(14) $$Specificity=\frac{TN}{TN+FP}.$$

The accuracy rate measures the proportion of classifications that a method generates correctly among all the instances in the data, regardless of the specific type of error (false positives or false negatives). The accuracy rate is defined in the equation as:

(15) $$Accuracy=\frac{TP+TN}{TP+TN+FP+FN}.$$

## 5. Experimental Results

### 5.1. Ablation Study

#### 5.1.1. Window-Based Self-Attention Module Selection

The proposed 2D-WinSpatt-Net method is inspired by the Swin Transformer [33,59]. Our method does not have a shifting window partition operation, which is different from the Swin Transformer. The core concept of the Swin Transformer involves the dynamic displacement of the window partition between successive self-attention blocks. Table 2 shows how the proposed 2D-WinSpatt-Net outperforms Swin Transformer V2 [59]. The AUC and the accuracy of the proposed 2D-WinSpatt-Net increase by 4% and 4%, respectively, compared to Swin Transformer V2.

#### 5.1.2. Lobal Spatial Attention Module Selection

**Figure 4:**
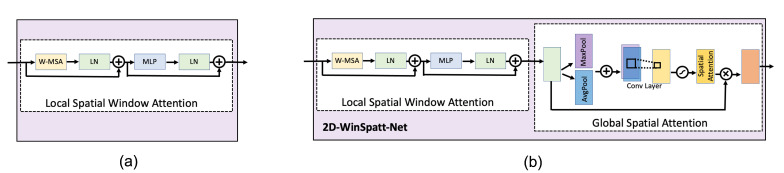
Ablation study using 20-s window length continuous wavelet transform (CWT) as input: (**a**) local spatial window attention module; (**b**) local spatial window attention module + global spatial attention module (the proposed 2D-WinSpatt-Net).

Figure 4 and Table 3 show the comparison of two methods. One method is using the local spatial window attention module only (abbreviated to window attention). The other is the proposed 2D-WinSpatt-Net, using a combination of the local spatial window attention module and the global spatial attention module. The suggested 2D-WinSpatt-Net works better than the Window Attention.

#### 5.1.3. Different Attention Methods

**Figure 5:**
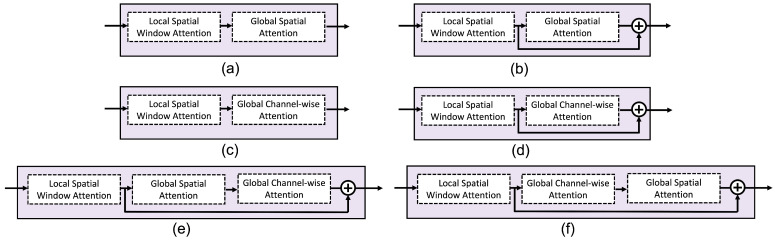
Ablation study of investigating different global attention modules: (**a**) Window Attention + Spatial Attention (the proposed 2D-WinSpatt-Net); (**b**) Window Attention + Spatial residual Attention; (**c**) Window Attention + Channel-wise Attention; (**d**) Window Attention + Channel-wise residual Attention; (**e**) Window Attention + (Spatial + Channel-wise) residual Attention; (**f**) Window Attention + (Channel-wise + Spatial) residual Attention.

The attention module is built based on window attention. In our experiments, we test different attention methods. Figure 5 shows details of the different attention modules built on window attention. Table 4 shows that the proposed 2D-WinSpatt-Net achieves better performance compared to other attention methods.

### 5.2. Comparisons

We compare the introduced 2D-WinSpatt-Net with six different DL techniques, including one- and two-dimensional convolutional neural networks. In light of the experimental outcomes presented in Table 2, the image-based 2D-WinSpatt-Net method using CWT as input achieves the best performance in diagnosing tetanus. The proposed 2D-WinSpatt-Net works better than our previous 2D-CNN-Transformer/8 method [13].

#### 5.2.1. Time Series Imaging

**Figure 6:**
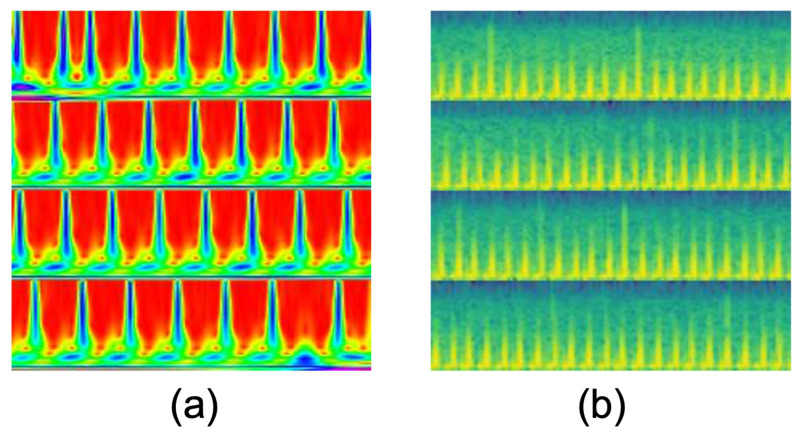
Comparing two time series imaging techniques: (**a**) Resized and stitched continuous wavelet transform (CWT) with a 20-s window length, resulting in 224 pixels ×224 pixels; (**b**) Resized and stitched log-spectrogram with a 60-s window length, resulting in 224 pixels ×224 pixels.

Figure 6 shows two types of time series imaging that are used as input in our 2D DL methods. We also compare the 2D-WinSpatt-Net using two different time series images as input. Table 2 shows the proposed 2D-WinSpatt-Net using resized and stitched CWT as input outperforms using resized and stitched log-spectrogram as input. The shorter resized and stitched CWT (20 s) as input achieve better performance than the resized and stitched log-spectrogram (60 s).

#### 5.2.2. Relation to Swin Transformer

We make a comparison with one representative baseline method, Swin Transformer V2 [59]. Both the proposed 2D-WinSpatt-Net and Swin Transformer V2 use window attention as an element of the network. The key concept of the Swin Transformer [33,59] is to shift the window partition between consecutive self-attention blocks. This approach, however, is not employed in the 2D-WinSpatt-Net. Table 2 shows that the proposed 2D-WinSpatt-Net achieves better performance, using resized and stitched CWT inputs with a window length of 20 s.

## 6. Discussion

The proposed 2D-WinSpatt-Net is a novel transformer-based method. It captures both the local and global attention information, which is based on the image patch token level. The local and global ECG information of the CWT boosts the classification of the tetanus severity level, which works better than our previous work, the 2D-CNN + Channel-wise Attention [12] and the 2D-CNN-Transformer/8 [13]. Moreover, the proposed 2D-WinSpatt-Net using resized and stitched 20-s window length CWT as input outperforms 2D-CNN and 2D-CNN + Dual attention using resized and stitched 60-s window length log-spectrograms as input. Furthermore, the proposed 2D-WinSpatt-Net (imaging in machine learning (ML)) beats 1D-CNN (non-imaging in ML). In addition, it outperforms the traditional ML—Random Forest using HRV time domain features [13].

We believe that the work presented here is the first to explore the benefits of using CWT-based transforms of wearable ECG signals as inputs for tetanus severity classification. Our results indicated that richer ECG time–frequency information could be captured using CWTs as compared to log-spectrograms—which significantly boosted downstream tetanus severity classification for all models explored. The CWT can provide a more informative time–frequency representation than the short-time Fourier transform, which is computed during a spectrogram. Without the need to coarsely window the signal, overcoming the uncertainty principle associated with computing the STFT, the CWT can obtain dynamic time–frequency resolutions directly from the entire ECG sequence through decomposing the signal into varying scales over time. For more information on the benefits of utilising CWT representations with respect to ECG classification, we refer the reader to Wang et al. (2021) [48] and Al et al. (2018) [60] for further reading.

We believe that the encoding of the key characteristics of our ECG signals in the time–frequency domain was better captured by the CWT methodology and therefore yielded a richer representation to our downstream 2D-WinSpatt-Net model. For example, the log-spectrogram, using the STFT, provides a uniform view of the time–frequency space, using a fixed window size, leading to a constant time–frequency resolution across all frequencies. In contrast, CWT is advantageous in that it provides a multi-resolution analysis—yielding good time resolution at high frequencies and good frequency resolution at low frequencies—which is achieved by varying the width of the wavelet. For instance, the QRS complex is a high-frequency event that lasts for a short duration, while the T-wave is a lower-frequency event spread over a longer time. Furthermore, ECGs are non-stationary signals; we are interested in characterising non-stationary properties, such as heart rate variability (HRV), morphological variations, baseline wander and artefacts. Due to this adaptability in resolution, the CWT can be better at handling non-stationary signals and can also be better at detecting transient events in an ECG signal, such as P-waves or T-waves, etc., especially when these events exist at different scales. Finally, the adaptability of the wavelet chosen often results in the better removal of common edge effects in ECG as compared to STFT, for example, when analysing ECG signals, where beginnings and endings (or abrupt changes) carry significant information which we want to characterize.

We use the discretised version of the CWT so that it can be implemented in a computational environment. Given its redundant nature, the CWT (especially in its discretized form) is preferred for signal analysis tasks where precise time–frequency localisation is crucial, such as in the detection of transient features in our ECG signal. The CWT offers a dense sampling in both the time and frequency domains, making it ideal for visualising our ECG signal and preserving the features that might be missed or inadequately represented by the coarser, dyadic scales of the DWT. Furthermore, the continuous nature of the CWT allows for flexibility in choosing scales, which can be important as we are interested in visualizing features that do not align necessarily with dyadic or other discrete scale sets.

Our methods could be applied to other infectious diseases, for example sepsis or dengue. The signal processing technology—time series imaging in a square shape—can be used in other fields, such as seismic signal analysis. The novel deep learning model—2D-WinSpatt-Net—can also apply to the image processing field and the medical imaging field.

In future work, we will explore various window durations of the raw ECG data to generate CWT, such as 60s, 50s, 40s, 30s, 20s, 10s and 5s window lengths. We would like to find the optimal shortest window length CWT which still maintains the accuracy of tetanus severity classification.

Currently, our work only uses ECG to classify tetanus severity. Normally, tetanus classification is dependent on respiratory features with or without added cardiovascular features.

The ultimate goal of our work is to develop a tetanus severity warning tool with the aim of improving clinical treatment outcomes and reducing the incidence of the disease [13]. By utilising ECG data collected through wearable sensors from patients, this tool will provide predictions on the severity of tetanus. It is designed to be applicable in both low-resource settings, where there is a scarcity of equipment and medical staff that affects patient care, and high-income settings [13], where inexperienced personnel may face challenges in managing tetanus due to the limited exposure to such cases. The implementation of this tool holds the potential to assist in clinical decision-making processes by preventing unnecessary admissions of mild cases to the ICU and reducing treatment delays for severe cases. By accurately predicting the severity of tetanus, it can contribute to optimizing resource allocation and improving patient outcomes.

## 7. Conclusions

We proposed a novel transformer-based method named 2D-WinSpatt-Net. This method has a dual attention mechanism, including local spatial window attention and global spatial attention. The experimental findings clearly indicate the superiority of our proposed 2D-WinSpatt-Ne over other advanced DL approaches when it comes to classifying tetanus severity levels. This novel deep learning framework has the potential to greatly enhance clinical care decision-making processes and facilitate the optimal allocation of limited healthcare resources, particularly in LMIC. Furthermore, the success of our method opens up possibilities for its application in similar infectious diseases, such as sepsis and dengue. In future work, we will aim to predict tetanus severity level on the future 5th day, using the tetanus patient ECG information on the 1st day at ICU. In addition, the 2D-WinSpatt-Net can be applied to classification tasks in different fields, including time series classification tasks. Overall, the proposed deep learning framework represents a significant advancement in the field and holds promise for addressing the challenges faced by healthcare systems in LMIC, ultimately contributing to better patient outcomes and resource utilisation. 

## Figures and Tables

**Table 1:** Train–valid–test split definition for the Ttnus dataset.

Dataset	30 ECG Time Series from Each ECG Example		
	Mild	Severe	Total Number
Training	2370	1860	4230
Validation	270	270	540
Test	360	210	570

**Table 2:** A quantitative analysis of the proposed 2D-WinSpatt-Net, utilizing resized and stitched continuous wavelet transform (CWT) with a 20-s window duration as input, compared to baseline methods that employ either resized and stitched log-spectrograms with a 60-s window duration or original 60-s window length ECG as input. The results are displayed as mean ± standard deviation, with the highest performance emphasised in bold.

**Method**	The 60-s Log-Spectrogram					**AUC**
	**F1 Score**	**Precision**	**Recall**	**Specificity**	**Accuracy**	
2D-CNN [12]	0.61 ± 0.14	0.68 ± 0.07	0.57 ± 0.19	0.85 ± 0.02	0.75 ± 0.07	0.72 ± 0.09
2D-CNN + Dual Attention [12]	0.65 ± 0.19	0.71 ± 0.17	0.61 ± 0.21	0.86 ± 0.09	0.76 ± 0.11	0.74 ± 0.13
2D-CNN + Channel-wise Attention [12]	0.79 ± 0.03	0.78 ± 0.08	0.82 ± 0.05	0.85 ± 0.08	0.84 ± 0.04	0.84 ± 0.03
2D-CNN-Transformer/8 [13]	0.82 ± 0.03	**0.94 ± 0.03**	0.73 ± 0.07	0.97 ± 0.02	0.88 ± 0.01	0.85 ± 0.03
Proposed 2D-WinSpatt-Net	0.75 ± 0.05	0.81 ± 0.02	0.70 ± 0.07	0.91 ± 0.00	0.83 ± 0.03	0.80 ± 0.04
	**The 20-s CWT**					
Swin Transformer V2	0.83 ± 0.03	0.93 ± 0.01	0.75 ± 0.04	**0.97 ± 0.01**	0.89 ± 0.01	0.86 ± 0.02
**Proposed 2D-WinSpatt-Net**	**0.88 ± 0.00**	0.92 ± 0.02	**0.85 ± 0.01**	0.96 ± 0.01	**0.93 ± 0.02**	**0.90 ± 0.00**
**Method**	**Image-Free Data Representation**					**AUC**
	**F1 Score**	**Precision**	**Recall**	**Specificity**	**Accuracy**	
1D-CNN [12]	0.65 ± 0.14	0.61 ± 0.05	0.77 ± 0.25	0.70 ± 0.13	0.73 ± 0.05	0.74 ± 0.08

**Table 3:** Analysing the effects of 2D-WinSpatt-Net: ablation studies with resized and stitched CWT input of 20-s window length. The results are displayed as mean ± standard deviation, with the highest performance emphasised in bold. The local spatial window attention module is abbreviated to window attention.

Method	The 20-s CWT: Resized & Stitched					AUC
	F1 Score	Precision	Recall	Specificity	Accuracy	
Window Attention	0.823 ± 0.033	**0.950 ± 0.012**	0.727 ± 0.050	**0.978 ± 0.006**	0.885 ± 0.018	0.852 ± 0.025
**Proposed 2D-WinSpatt-Net**	**0.884 ± 0.003**	0.924 ± 0.016	**0.848 ± 0.011**	0.959 ± 0.010	**0.926 ± 0.020**	**0.903 ± 0.002**

**Table 4:** A quantitative evaluation of the proposed 2D-WinSpatt-Net and the baseline methods using resized and stitched continuous wavelet transform (CWT) with a window duration of 20-s. The results are displayed as mean ± standard deviation, with the highest performance emphasised in bold. The local spatial window attention module is abbreviated to window attention. The global spatial attention module is abbreviated to window attention.

**Method**	The 20-s CWT: Resized & Stitched					**AUC**
	**F1 Score**	**Precision**	**Recall**	**Specificity**	**Accuracy**	
Window Attention + Spatial residual Attention	0.87 ± 0.01	0.93 ± 0.00	0.81 ± 0.01	0.97 ± 0.00	0.91 ± 0.00	0.89 ± 0.01
**Window Attention + Spatial Attention (2D-WinSpatt-Net)**	**0.88 ± 0.00**	0.92 ± 0.02	**0.85 ± 0.01**	0.96 ± 0.01	**0.93 ± 0.02**	**0.90 ± 0.00**
Window Attention + Channel-wise residual Attention	0.83 ± 0.01	0.93 ± 0.02	0.74 ± 0.03	0.97 ± 0.01	0.89 ± 0.01	0.86 ± 0.01
Window Attention + Channel-wise Attention	0.83 ± 0.02	**0.95 ± 0.00**	0.74 ± 0.03	**0.98 ± 0.00**	0.89 ± 0.01	0.86 ± 0.01
Window Attention + (Channel-wise + Spatial) residual Attention	0.85 ± 0.03	0.94 ± 0.02	0.78 ± 0.06	0.97 ± 0.01	0.90 ± 0.01	0.88 ± 0.02
Window Attention + (Spatial + Channel-wise) residual Attention	0.88 ± 0.01	0.92 ± 0.02	**0.85 ± 0.01**	0.95 ± 0.01	0.92 ± 0.01	**0.90 ± 0.00**

## Data Availability

Not applicable.

## References

[1] Thwaites C.L., Yen L.M., Glover C., Tuan P.Q., Nga N.T.N., Parry J., Loan H.T., Bethell D., Day N.P.J., White N.J. (2006). Predicting the clinical outcome of tetanus: The tetanus severity score. Trop. Med. Int. Health.

[2] Yen L.M., Thwaites C.L. (2019). Tetanus. Lancet.

[3] Thuy D.B., Campbell J.I., Thanh T.T., Thuy C.T., Loan H.T., Hao N.V., Minh Y.L., Tan L.V., Boni M.F., Thwaites C.L. (2017). Tetanus in southern Vietnam: Current situation. Am. J. Trop. Med. Hyg..

[4] Thwaites C. (2017). Botulism and tetanus. Medicine.

[5] Disease Factsheet about Tetanus. https://www.ecdc.europa.eu/en/tetanus/facts.

[6] The Importance of Diagnostic Tests in Fighting Infectious Diseases. https://www.lifechanginginnovation.org/medtech-facts/importance-diagnostic-tests-fighting-infectious-diseases.html.

[7] Van H.M.T., Van Hao N., Quoc K.P.N., Hai H.B., KhoaLe D.V., Yen L.M., Nhat P.T.H., Duong H.T.H., Thuy D.B., Zhu T. (2021). Vital sign monitoring using wearable devices in a Vietnamese intensive care unit. BMJ Innov..

[8] Mahieu R., Reydel T., Maamar A., Tadié J.M., Jamet A., Thille A.W., Chudeau N., Huntzinger J., Grangé S., Beduneau G. (2017). Admission of tetanus patients to the ICU: A retrospective multicentre study. Ann. Intensive Care.

[9] Hung T.M., Van Hao N., Yen L.M., McBride A., Dat V.Q., van Doorn H.R., Loan H.T., Phong N.T., Llewelyn M.J., Nadjm B. (2022). Direct Medical Costs of Tetanus, Dengue, and Sepsis Patients in an Intensive Care Unit in Vietnam. Front. Public Health.

[10] Hung T.M., Clapham H.E., Bettis A.A., Cuong H.Q., Thwaites G.E., Wills B.A., Boni M.F., Turner H.C. (2018). The estimates of the health and economic burden of dengue in Vietnam. Trends Parasitol..

[11] Joshi M., Ashrafian H., Aufegger L., Khan S., Arora S., Cooke G., Darzi A. (2019). Wearable sensors to improve detection of patient deterioration. Expert Rev. Med. Devices.

[12] Lu P., Ghiasi S., Hagenah J., Hai H.B., Hao N.V., Khanh P.N.Q., Khoa L.D.V., Thwaites L., Clifton D.A., VITAL Consortium (2022). Classification of Tetanus Severity in Intensive-Care Settings for Low-Income Countries Using Wearable Sensing. Sensors.

[13] Lu P., Wang C., Hagenah J., Ghiasi S., Zhu T., Thwaites L., Clifton D.A., VITAL consortium (2022). Improving Classification of Tetanus Severity for Patients in Low-Middle Income Countries Wearing ECG Sensors by Using a CNN-Transformer Network. IEEE Trans. Biomed. Eng..

[14] Duong H.T.H., Tadesse G.A., Nhat P.T.H., Van Hao N., Prince J., Duong T.D., Kien T.T., Nhat L.T.H., Van Tan L., Pugh C. (2020). Heart rate variability as an indicator of autonomic nervous system disturbance in tetanus. Am. J. Trop. Med. Hyg..

[15] Cygankiewicz I., Zareba W. (2013). Heart rate variability. Handb. Clin. Neurol..

[16] Lombardi F., Malliani A. (1996). Heart rate variability: Standards of measurement, physiological interpretation and clinical use. Task Force of the European Society of Cardiology and the North American Society of Pacing and Electrophysiology. Circulation.

[17] Bolanos M., Nazeran H., Haltiwanger E. Comparison of heart rate variability signal features derived from electrocardiography and photoplethysmography in healthy individuals. Proceedings of the 2006 International Conference of the IEEE Engineering in Medicine and Biology Society.

[18] Tadesse G.A., Javed H., Thanh N.L.N., Thi H.D.H., Thwaites L., Clifton D.A., Zhu T. (2020). Multi-modal diagnosis of infectious diseases in the developing world. IEEE J. Biomed. Health Inform..

[19] Kiyasseh D., Tadesse G.A., Thwaites L., Zhu T., Clifton D. (2020). Plethaugment: Gan-based ppg augmentation for medical diagnosis in low-resource settings. IEEE J. Biomed. Health Inform..

[20] Ghiasi S., Zhu T., Lu P., Hagenah J., Khanh P.N.Q., Hao N.V., Thwaites L., Clifton D.A., Vital Consortium (2022). Sepsis Mortality Prediction Using Wearable Monitoring in Low-Middle Income Countries. Sensors.

[21] Tadesse G.A., Zhu T., Le Nguyen Thanh N., Hung N.T., Duong H.T.H., Khanh T.H., Van Quang P., Tran D.D., Yen L.M., Van Doorn R. (2020). Severity detection tool for patients with infectious disease. Healthc. Technol. Lett..

[22] Ullah A., Anwar S.M., Bilal M., Mehmood R.M. (2020). Classification of arrhythmia by using deep learning with 2-D ECG spectral image representation. Remote Sens..

[23] Zihlmann M., Perekrestenko D., Tschannen M. Convolutional recurrent neural networks for electrocardiogram classification. Proceedings of the 2017 Computing in Cardiology (CinC).

[24] Diker A., Cömert Z., Avcı E., Toğaçar M., Ergen B. A novel application based on spectrogram and convolutional neural network for ecg classification. Proceedings of the 2019 1st International Informatics and Software Engineering Conference (UBMYK).

[25] Liu G., Han X., Tian L., Zhou W., Liu H. (2021). ECG quality assessment based on hand-crafted statistics and deep-learned S-transform spectrogram features. Comput. Methods Programs Biomed..

[26] Creagh A.P., Simillion C., Bourke A.K., Scotland A., Lipsmeier F., Bernasconi C., van Beek J., Baker M., Gossens C., Lindemann M. (2020). Smartphone-and smartwatch-based remote characterisation of ambulation in multiple sclerosis during the two-minute walk test. IEEE J. Biomed. Health Inform..

[27] Tutuko B., Nurmaini S., Tondas A.E., Rachmatullah M.N., Darmawahyuni A., Esafri R., Firdaus F., Sapitri A.I. (2021). AFibNet: An implementation of atrial fibrillation detection with convolutional neural network. BMC Med. Inform. Decis. Mak..

[28] Kiranyaz S., Avci O., Abdeljaber O., Ince T., Gabbouj M., Inman D.J. (2021). 1D convolutional neural networks and applications: A survey. Mech. Syst. Signal Process..

[29] Wu Y., Yang F., Liu Y., Zha X., Yuan S. (2018). A comparison of 1-D and 2-D deep convolutional neural networks in ECG classification. arXiv.

[30] Vaswani A., Shazeer N., Parmar N., Uszkoreit J., Jones L., Gomez A.N., Kaiser Ł., Polosukhin I. Attention is all you need. Proceedings of the Advances in Neural Information Processing Systems 30 (NIPS 2017).

[31] Han K., Wang Y., Chen H., Chen X., Guo J., Liu Z., Tang Y., Xiao A., Xu C., Xu Y. (2022). A survey on vision transformer. IEEE Trans. Pattern Anal. Mach. Intell..

[32] Dosovitskiy A., Beyer L., Kolesnikov A., Weissenborn D., Zhai X., Unterthiner T., Dehghani M., Minderer M., Heigold G., Gelly S. (2020). An image is worth 16x16 words: Transformers for image recognition at scale. arXiv.

[33] Liu Z., Lin Y., Cao Y., Hu H., Wei Y., Zhang Z., Lin S., Guo B. Swin transformer: Hierarchical vision transformer using shifted windows. Proceedings of the IEEE/CVF International Conference on Computer Vision.

[34] Touvron H., Cord M., Douze M., Massa F., Sablayrolles A., Jégou H. Training data-efficient image transformers & distillation through attention. Proceedings of the International Conference on Machine Learning.

[35] Hinton G., Vinyals O., Dean J. (2015). Distilling the knowledge in a neural network. arXiv.

[36] Han K., Xiao A., Wu E., Guo J., Xu C., Wang Y. Transformer in transformer. Proceedings of the Advances in Neural Information Processing Systems 34 (NeurIPS 2021).

[37] Hatamizadeh A., Tang Y., Nath V., Yang D., Myronenko A., Landman B., Roth H.R., Xu D. Unetr: Transformers for 3d medical image segmentation. Proceedings of the IEEE/CVF Winter Conference on Applications of Computer Vision.

[38] Chen J., Lu Y., Yu Q., Luo X., Adeli E., Wang Y., Lu L., Yuille A.L., Zhou Y. (2021). Transunet: Transformers make strong encoders for medical image segmentation. arXiv.

[39] Zhao C., Droste R., Drukker L., Papageorghiou A.T., Noble J.A. Visual-Assisted Probe Movement Guidance for Obstetric Ultrasound Scanning Using Landmark Retrieval. Proceedings of the International Conference on Medical Image Computing and Computer-Assisted Intervention.

[40] Zhang J., Li C., Liu G., Min M., Wang C., Li J., Wang Y., Yan H., Zuo Z., Huang W. (2022). A CNN-transformer hybrid approach for decoding visual neural activity into text. Comput. Methods Programs Biomed..

[41] Wu H., Chen S., Chen G., Wang W., Lei B., Wen Z. (2022). FAT-Net: Feature adaptive transformers for automated skin lesion segmentation. Med Image Anal..

[42] Gong Y., Chung Y.A., Glass J. (2021). AST: Audio Spectrogram Transformer. arXiv.

[43] Park S., Jeong Y., Lee T. Many-to-Many Audio Spectrogram Transformer: Transformer for Sound Event Localization and Detection. Proceedings of the Detection and Classification of Acoustic Scenes and Events 2021.

[44] Kong Q., Xu Y., Wang W., Plumbley M.D. (2020). Sound event detection of weakly labelled data with CNN-transformer and automatic threshold optimization. IEEE/ACM Trans. Audio Speech Lang. Process..

[45] Byeon Y.H., Kwak K.C. (2019). Pre-configured deep convolutional neural networks with various time-frequency representations for biometrics from ECG signals. Appl. Sci..

[46] Virtanen P., Gommers R., Oliphant T.E., Haberland M., Reddy T., Cournapeau D., Burovski E., Peterson P., Weckesser W., Bright J. (2020). SciPy 1.0: Fundamental Algorithms for Scientific Computing in Python. Nat. Methods.

[47] Addison P.S., Walker J., Guido R.C. (2009). Time–frequency analysis of biosignals. IEEE Eng. Med. Biol. Mag..

[48] Wang T., Lu C., Sun Y., Yang M., Liu C., Ou C. (2021). Automatic ECG classification using continuous wavelet transform and convolutional neural network. Entropy.

[49] Torrence C., Compo G.P. (1998). A practical guide to wavelet analysis. Bull. Am. Meteorol. Soc..

[50] Lilly J.M., Olhede S.C. (2008). Higher-order properties of analytic wavelets. IEEE Trans. Signal Process..

[51] Khandelwal S., Wickström N. (2018). Novel methodology for estimating Initial Contact events from accelerometers positioned at different body locations. Gait Posture.

[52] Banerjee S., Mitra M. (2013). Application of cross wavelet transform for ECG pattern analysis and classification. IEEE Trans. Instrum. Meas..

[53] Abry P. (1997). *Ondelettes et turbulences: Multirésolutions, Algorithmes de Décomposition, Invariance d’échelle et Signaux de Pression*, Diderot éd.

[54] Hendrycks D., Gimpel K. (2016). Gaussian error linear units (gelus). arXiv.

[55] Ba J.L., Kiros J.R., Hinton G.E. (2016). Layer normalization. arXiv.

[56] Hu J., Shen L., Sun G. Squeeze-and-excitation networks. Proceedings of the IEEE Conference on Computer Vision and Pattern Recognition.

[57] Woo S., Park J., Lee J.Y., Kweon I.S. CBAM: Convolutional block attention module. Proceedings of the European Conference on Computer Vision (ECCV).

[58] Bradley A.P. (1997). The use of the area under the ROC curve in the evaluation of machine learning algorithms. Pattern Recognit..

[59] Liu Z., Hu H., Lin Y., Yao Z., Xie Z., Wei Y., Ning J., Cao Y., Zhang Z., Dong L. Swin transformer v2: Scaling up capacity and resolution. Proceedings of the IEEE/CVF Conference on Computer Vision and Pattern Recognition.

[60] Al Rahhal M.M., Bazi Y., Al Zuair M., Othman E., BenJdira B. (2018). Convolutional neural networks for electrocardiogram classification. J. Med. Biol. Eng..

